# Treatment and Prevention of Opioid Use Disorder: Challenges and Opportunities

**DOI:** 10.1146/annurev-publhealth-040617-013526

**Published:** 2017-12-22

**Authors:** Dennis McCarty, Kelsey C. Priest, P. Todd Korthuis

**Affiliations:** 1 Oregon Health & Science University–Portland State University School of Public Health, Portland, Oregon 97239, USA; 2 Department of Psychiatry, School of Medicine, Oregon Health & Science University, Portland, Oregon 97239, USA; 3 MD/PhD Program, School of Medicine, Oregon Health & Science University, Portland, Oregon 97239, USA; 4 Addiction Medicine Section, Department of Medicine, School of Medicine, Oregon Health & Science University, Portland, Oregon 97239, USA

**Keywords:** opioid use disorder, treatment for opioid use disorder, opioid agonist therapy, opioid antagonist therapy, opioid overdose prevention

## Abstract

Treatment for opioid use disorder in the United States evolved in response to changing federal policy and advances in science. Inpatient care began in 1935 with the US Public Health Hospitals in Lexington, Kentucky, and Fort Worth, Texas. Outpatient clinics emerged in the 1960s to provide aftercare. Research advances led to opioid agonist and opioid antagonist therapies. When patients complete opioid withdrawal, return to use is often rapid and frequently deadly. US and international authorities recommend opioid agonist therapy (i.e., methadone or buprenorphine). Opioid antagonist therapy (i.e., extended-release naltrexone) may also inhibit return to use. Prevention efforts emphasize public and prescriber education, use of prescription drug monitoring programs, and safe medication disposal options. Overdose education and naloxone distribution promote access to rescue medication and reduce opioid overdose fatalities. Opioid use disorder prevention and treatment must embrace evidence-based care and integrate with physical and mental health care.

## INTRODUCTION

Opioid use is widespread, and treatment access is limited. Data from the 2015 National Survey on Drug Use and Health suggest that among individuals 12 years of age and older, an estimated 97.5 million individuals (36.4% of the population) used prescription opioids in the past year, 12.5 million (4.7% of the population) misused prescription opioids, 2 million (0.8% of the population) individuals using prescription opioids met the criteria for diagnosis of an opioid use disorder, and 822,000 received treatment (38). An estimated 828,000 people used heroin, and most (72%) also used prescription opioids (38). For more detail on the epidemiology of opioid use, see the 2015 *Annual Review of Public Health* article by Kolodny et al. (54).

Patient and physician essays in the January 2017 issue of *Health Affairs* illustrate the challenges of receiving and providing appropriate treatment for opioid use disorder. The patient described his ordeal of withdrawing from long-term opioid use because his physician did not know how to manage his opioid withdrawal (82). A physician, conversely, lamented that his patient returned to heroin injection despite a prescription for buprenorphine, “I suddenly felt like I was his enabler—and his dealer” (p. 187); after reflection, the physician recognized that the patient had a chronic disease that required long-term medical management and renewed his relationship with the patient (60).

The US Food and Drug Administration (FDA) has approved two opioid agonist medications for the treatment of opioid use disorders: methadone (a Schedule II controlled substance and a full opioid agonist) and buprenorphine (a Schedule III controlled substance and a partial opioid agonist). All practitioners who prescribe controlled substances are required to register with the Drug Enforcement Administration (DEA). Prescribers who treat opioid use disorder, however, have additional requirements. To dispense methadone for opioid withdrawal management or for long-term care, providers are required to register with the Drug Enforcement Administration (DEA) and supervise patients in a federally licensed opioid treatment program. The opioid treatment program regulations are demanding, and few physicians seek to meet the requirements. To prescribe buprenorphine, practitioners (i.e., physicians, physician assistants, and nurse practitioners) are required to meet the Substance Abuse and Mental Health Services Administration (SAMHSA) training and experience requirements, be DEA registered and approved, receive a unique DEA identification number, comply with record-keeping requirements, and limit the size of their patient panel. [The initial limit is 30 patients. Practitioners can request an increase to 100 patients after 1 year of experience and increase their panel up to 275 patients if they are credentialed in addiction medicine or addiction psychiatry or if they agree to additional practice requirements (27). Non-physician prescribers have additional limits.]

The treatment of opioid use disorder is further complicated because this condition is often associated with serious medical comorbidities that require relatively intensive medical intervention (35, 84). Many patients, moreover, have histories of incarceration, unemployment, homelessness, and psychiatric comorbidities and require social service interventions that are not usually found in the medical system (77). This article focuses on treatment models, approaches for addressing opioid use disorder in primary care and specialty care settings, and interventions to prevent overdose and reduce health risks.

## DEVELOPMENT OF OPIOID TREATMENT MODELS

Opioid use disorder treatment models evolved in response to changing federal policy and scientific discovery. Musto’s (68) and Courtwright’s (16, 17) historical analyses of narcotic control and opioid use recount the unregulated use of opiates in the first decades of the twentieth century; the sympathetic use of morphine to address iatrogenic addiction; changes in state, federal, and international policies to control drug use; and addiction treatment services in the years prior to and immediately following passage of the Harrison Narcotic Act of 1914 (P.L. 63–223).

The Harrison Narcotic Act required physicians and pharmacists who handled opium and cocaine to register with the US Department of the Treasury, pay a tax, and keep records of the narcotics dispensed. The Treasury Department interpreted the statute as a prohibition against physicians prescribing narcotics to treat narcotic addiction; a 1919 US Supreme Court decision [*Webb v. United States*] upheld the Treasury Department’s interpretation (68). Subsequently, communities developed morphine dispensaries modeled on services for tuberculosis and sexually transmitted diseases (68). Aggressive federal prosecution closed the dispensaries because they violated the prohibition on the use of narcotics to treat narcotic addiction. Arrest and incarceration replaced clinical services as the preferred intervention for drug use and addiction (39, 96a).

### Public Health Hospitals

In 1929, federal legislation authorized construction of two Narcotic Farms. The farms opened in Lexington, Kentucky, in 1935 (for individuals living east of the Mississippi River) and in Fort Worth, Texas, in 1938 (for individuals living west of the Mississippi River) to treat narcotic addiction and to conduct research. When the Lexington facility opened, Congress changed the official name to the US Public Health Hospitals under the joint management of the US Public Health Service and the US Bureau of Prisons. The “hospitals” served volunteers and individuals convicted of federal drug crimes. Based on treatment standards at that moment in time, moral therapy guided inpatient care: healthy living and hard work in a rural setting (13). Treatment included withdrawal management, a drug-free environment, psychotherapy, and supervised activity (e.g., work, education); the hospitals also provided medical and dental care (62). The recommended length of stay was four to six months, but most volunteers completed withdrawal and left against medical advice; federal prisoners were required to complete their sentences (62). There was no supervision or aftercare post release, and return to use was common. The hospitals were closed in 1974, and the facilities became minimum-security federal correctional institutions.

### Community-Based Outpatient Services

The 1966 Narcotic Addict Rehabilitation Act (NARA; P.L. 93–281) was modeled on legislation in California and New York that committed individuals convicted of drug crimes to community treatment and supervision with frequent urine drug screening (10). Extending the civil commitment, community supervision, and aftercare practices to federal courts created incentives for treatment and expanded access to the Public Health Hospitals (62). In lieu of prosecution, individuals charged with a federal drug offense could accept a 36-month civil commitment, complete 4–6 months at the Public Health Hospitals and receive ongoing supervision and aftercare following hospital release (62). The legislation also authorized civil commitments (of up to 10 years) for offenders convicted of a federal drug offense and extended the civil commitment opportunity to individuals who were not charged with an offense (a user or family member could petition for a hospital commitment and community supervision post-release) (62). The Lexington and Fort Worth hospitals provided the inpatient care, and outpatient services were developed in larger communities. Services were not limited to persons with opioid use disorder, but most participants were dependent on heroin and other opioids.

The NARA also provided federal awards to states and communities to provide community-based supervision and aftercare (10, 62). Besteman (10) recounts that the NARA implementation plan envisioned a slow, gradual development of caseloads and training for community-based clinicians; in practice, however, federal courts committed offenders to care before providers were ready, forcing community-based services to develop quickly. The clinics focused on achieving abstinence and became identified as outpatient drug-free treatment. The federal funding for outpatient care and federal training for drug counselors and professionals were the first investments in community-based care for opioid use disorder (61).

### Therapeutic Communities

Long-term residential care in community settings emerged in the late 1950s and early 1960s as therapeutic communities. Synanon, the prototypic therapeutic community, used staff in recovery and encounter groups to confront residents (many had long histories of crime and incarceration), challenging them to be citizens of and responsible to the community by living without the use of drugs (39, 41). Participants often lacked the training and skills required to live without drugs. Long lengths of stay were expected; rehabilitation required 15–24 months to modify dysfunctional behaviors, eliminate criminal thinking and behavior, and develop skills of daily living (e.g., employment, self-honesty, responsibility) (18). Confrontations and interventions in therapeutic communities were often dehumanizing, and many residents left quickly. The dropout rate (71%), however, was similar to dropout rates in outpatient programs (74%) and reflected (*a*) an open-door policy by which residents could enter or leave at any time, (*b*) minimal admission criteria to encourage treatment entry, and (*c*) the intensity of therapeutic community practices (18). Overtime practices moderated, and lengths of stay contracted. The legacy of tough love and verbal abuse continues within the drug treatment services provided in penal institutions, often called therapeutic communities behind walls.

### Opioid Agonist Therapies

Opioid agonists attach to and fully activate (e.g., morphine, methadone) or partially activate (e.g., buprenorphine) μ-opioid receptors preventing opioid withdrawal. Morphine dispensaries were the first approach to opioid agonist therapy. The concept re-emerged in the 1960s with the use of methadone (45). Methadone treatment began as research and was quietly tolerated despite the prohibition against the use of narcotics for the treatment of narcotic addiction. Initial work in New York City (23, 24) documented that methadone administered orally reduced opioid craving and opioid use and contributed to increased employment, improved health, and reduced criminal activity (42, 57). New York City rapidly expanded access to methadone research programs, and other locations began their own methadone research programs.

Although the data were strong, the long legacy of federal prohibition of agonist therapy left federal regulators skeptical and even hostile; regulations were imposed to restrict treatment entry, methadone dosage, and duration of care to discourage the services (42). In 1971, however, the Special Action Office for Drug Abuse Prevention (SAODAP) opened under the leadership of Jerry Jaffe. SAODAP worked closely with the FDA and revised methadone regulations to encourage expanded services while restricting the use of methadone to DEA approved narcotic treatment programs (now known as opioid treatment programs) that provide a full range of counseling, education, and support services (42). Patients, initially, attend a clinic daily for dispensed methadone. After stabilization, some patients may receive take-home medication, and daily attendance is no longer required.

In the 1970s and 1980s, methadone programs grew and expanded while adhering to strict DEA and FDA guidelines dictating whom could be served, how they could be served, and where they could be served. A 1995 Institute of Medicine review of methadone services and regulatory structures recommended more programmatic flexibility as long as the programs met state or national accreditation standards (40). SAMHSA assumed regulatory responsibility for opioid treatment programs in 2000 and established accreditation standards.

The development and approval of buprenorphine (a partial opioid agonist) for the treatment of opioid use disorder extended contemporary treatment options. Initial tests on buprenorphine (conducted within the Public Health Hospital in Lexington, Kentucky) suggested that this compound could be clinically effective for opioid withdrawal and maintenance (44). Subsequent research documented its effectiveness and increased safety (partial agonists have reduced capacity to suppress respiration), but FDA approval was required and statutory amendments were needed to permit providers to prescribe an opioid agonist therapy (43). The Drug Addiction Treatment Act of 2000 (known as DATA 2000) provided legislative approval for the prescription of Schedule III, IV, or V controlled substances (see the sidebar titled Controlled Substances Schedules for more information on how controlled substances are categorized and scheduled). DATA 2000 physicians must complete eight hours of training and register with the DEA (receiving a DATA waiver). Nurse practitioners and physician assistants require 24 hours of training before they can receive a waiver. Buprenorphine (Schedule III) is the only medication that has FDA approval for use under DATA 2000. There are three FDA-approved formulations of buprenorphine: sublingual buprenorphine, a combination of sublingual buprenorphine and naloxone, and a six-month buprenorphine implant. An extended-release injection formulation is also in development. Additional regulations constrain the use of buprenorphine.

## CONTROLLED SUBSTANCES SCHEDULES

The scheduling system is the US federal regulatory mechanism for categorizing controlled substances based on acceptable medical use and the potential for abuse or dependency (22). The Controlled Substances Act of 1970, section 812, created five categories on the basis of perceived risk of diversion, abuse, and misuse:

- Schedule I controlled substances have no currently accepted medical use or a lack of safety for use under medical supervision (e.g., heroin, cannabis);
- Schedule II/IIN controlled substances have a high abuse potential, which may lead to severe psychological or physical dependence (e.g., II: methadone, hydromorphone; IIN: amphetamine, methamphetamine);
- Schedule III/IIIN controlled substances have a lower abuse potential than do the substances categorized as Schedule I or II and may lead to moderate or low physical dependence (e.g., III: buprenorphine, acetaminophen with codeine; IIIN: ketamine, anabolic steroids);
- Schedule IV controlled substances have a lower abuse potential than do Schedule III substances (e.g., lorazepam, clonazepam);
- Schedule V controlled substances have a lower abuse potential than do Schedule IV substances and include pharmaceuticals containing limited quantities of certain narcotics (e.g., promethazine with codeine, ezogabine)

A complete list of all scheduling actions, controlled substances, and regulated chemicals can be accessed at https://www.deadiversion.usdoj.gov/schedules/orangebook/orangebook.pdf

The Controlled Substances Act [section 303(g)(2)] limits the number of patients with opioid use disorder that a physician is allowed to treat with buprenorphine. The initial regulations limited prescribers’ panels to a maximum of 30 patients, and after a year, prescribers could request an increase to 100 patients. SAMHSA and the Department of Health and Human Services amended regulations in July 2016; eligible providers may now request an increase to 275 patients. The 2016 Comprehensive Addiction Recovery Act (CARA) expanded the practitioners who are authorized to prescribe buprenorphine to include physician assistants and nurse practitioners upon the completion of opioid use disorder treatment training and DEA and SAMHSA registration.

### Opioid Antagonist Therapy

Despite the documented efficacy of opioid agonist therapy, public and political skepticism and stigma persist. The 1972 Drug Abuse Office and Treatment Act authorizing SAODAP required the Office to develop non-addictive antagonist medications to treat heroin addiction (46). Opioid antagonist therapies block the μ-opioid receptor and prevent the euphoric and physiological effects of opioids; the medications have no addictive or dependence potential and are not controlled substances (81). Initial tests documented proof of concept, but adverse side effects limited clinical value; naltrexone was synthesized as a promising alternative opioid antagonist without serious side effects (76). The National Institute on Drug Abuse, in collaboration with the patent-holding pharmaceutical company, led the developmental effort (46). The FDA approved an oral naltrexone tablet as a treatment for opioid dependence in 1984. Subsequent research found that oral naltrexone was effective as a treatment for alcohol dependence, and the FDA approved that indication.

Despite the appeal of an opioid antagonist therapy and the documentation of efficacy, oral naltrexone has poor effectiveness in clinical use because daily dosing is required. A systematic review of 13 randomized controlled trials with 1,158 study participants found that study outcomes among individuals randomized to oral naltrexone were equivalent to outcomes among individuals assigned to placebo or to no medication (66). To address the poor medication adherence, an extended release injectable formulation of naltrexone was developed (34) and, after testing, received FDA approval as a treatment for alcohol use disorder in 2006 and as a medication to prevent opioid relapse in 2010. The primary clinical trial testing extended-release naltrexone for opioid use disorder, conducted in Russia where opioid agonist treatment with methadone is illegal, documented enhanced opioid abstinence and reduced opioid cravings in study participants receiving active medication relative to participants receiving placebo (58). Clinical trials comparing the effectiveness of buprenorphine versus injectable naltrexone are currently under way.

## TREATMENT ACCESS

The 2016 National Survey of Substance Abuse Treatment Services (N-SSATS), an annual census of specialty addiction treatment programs (clinics that rely on private insurance or self-pay are underrepresented), included data from 14,399 facilities; 82% (*n* = 11,836) provided outpatient care; 24% (*n* = 3,469) offered residential care for detoxification (*n* = 954), short-term care (30 days or less; *n* = 1,816), or long-term care (more than 30 days; *n* = 2,814); and 5% (*n* = 751) were hospitals with specialty detoxification and treatment beds (84a). Most specialty addiction treatment programs treated opioid use disorder, but only a subset provided either opioid agonist therapy (methadone or buprenorphine) or opioid antagonist therapy (extended-release naltrexone) to support long-term recovery and to prevent a return to use. Ongoing opioid agonist therapy with methadone was available primarily in outpatient programs accredited and registered as opioid treatment programs (*n =* 1,283); some opioid treatment programs also offered buprenorphine (*n =* 789) and 337 prescribed extended-release naltrexone (84a). In non-opioid treatment programs, 3,101 facilities provided buprenorphine and 2,691 facilities provided extended-release naltrexone (84a). Analyses suggest a substantial increase in program use of opioid agonist and antagonist therapies over time (84a). Note, these reports do not include patients receiving buprenorphine prescriptions in primary care practices.

Despite the increasing availability of opioid agonist and antagonist pharmacotherapies to support recovery, program surveys and SAMHSA data suggest that only a minority of patients with opioid use disorder receive medication support. A survey of 345 addiction treatment facilities reported that 34% of the facilities used pharmacotherapy for opioid use disorder (52), and an analysis of programs participating in the National Drug Abuse Treatment Clinical Trials Network found that only 10% of opioid use disorder patients received opioid agonist or opioid antagonist therapy (53). Frequently cited barriers to the use of agonist and antagonist medication include patients and families who request drug-free treatment, persistent expectations of abstinence as the only appropriate treatment outcome, staff resistance to the use of medications, and the cost of the medications; many addiction treatment centers, moreover, do not have prescribers on staff (51). Barriers to the use of extended-release naltrexone included the complexity of ordering and using the medication, health plan policies requiring prior authorization and utilization review, requirements to fail first at other therapies, the need for patients to be opioid free for 7–10 days prior to injection, the lack of physician continuity of care, and cultures resistant to the use of medication (4).

## TREATMENT UTILIZATION

SAMHSA requires states to report all publicly funded admissions and includes the data in the Treatment Episode Data Set (TEDS). The 2015 TEDS report included more than 1.5 million total admissions; 34% (*n =* 526,686) reported opioids as the primary drug of abuse (heroin = 401,743; other opioids = 124,943) (84b). Admissions for opioid use disorder increased 58% relative to 2005 (*n* = 332,401), and the proportion of patients reporting primary use of opioids other than heroin increased 75% between 2005 and 2015 compared with a 53% increase in admissions reporting heroin as their primary drug (84b). About one-third of the patients using heroin (32%) and patients using other opioids (34%) began use of opioids at 18 years of age or younger; most reported prior treatment admissions (heroin = 78%; other opioids = 60%) and only a minority had treatment plans that included opioid agonist or antagonist therapy (heroin = 37%; other opioids = 31%) (84b). Medicaid (50% for both groups) and other government resources (heroin = 30%; other opioids = 24%) were the primary payers for care (84b). Injection was the primary method of use for patients using heroin (69%), whereas oral was the dominant method of use for patients using other opioids (60%); most patients (heroin = 82%; other opioids = 82%) were white non-Hispanic and a minority of patients were women (heroin = 36%; other opioids = 48%) (84b).

## RETURN TO USE

A primary challenge when addressing opioid use disorder is the difficulty of tapering patients off of opioids and the rate at which individuals return to use. A 1926 report to Britain’s Ministry of Health examined the medical aspects of morphine and heroin addiction and concluded that the prognosis for cure was poor: “Relapse, sooner or later, appears to be the rule and permanent cure the exception” (65, p. 14). Similarly, the first annual report from the Lexington Public Health Hospital noted that most voluntary patients left before treatment was completed; data from 1943 documented low rates of abstinence at 6 months post-discharge and noted that individuals assigned to the hospital as a condition of probation (27% abstinent) or parole (31% abstinent) were more likely to be abstinent than voluntary patients (13% abstinent) (62). Community aftercare services under NARA did not improve outcomes; abstinence rates at six months post-discharge were around 14% (62). Abstinence was uncommon among graduates from therapeutic communities as well, although outcomes improved with subsequent readmissions (18). Longitudinal analyses of 581 individuals committed to treatment under California’s Civil Addiction Program between 1962 and 1964 found elevated rates of mortality and continued opioid use (37). Among the individuals who were still living 33 years after civil commitment (23% of the original cohort), 56% provided urine samples that were negative for opioids, 14% were incarcerated, and 30% had urine that tested positive for opioids or refused to provide a sample (37).

Contemporary research continues to find rapid rates of return to use among patients not using opioid agonist or antagonist therapy after withdrawal and cessation of opioids. The Prescription Opioid Addiction Treatment Study recruited prescription opioid users diagnosed with opioid use disorder and seeking treatment. Study participants received a brief buprenorphine stabilization (for 2 weeks) and an opioid taper (for 2 weeks) with 8 weeks of follow-up. Study participants who were unable to taper or returned to use during the follow-up period received an extended buprenorphine stabilization (for 12 weeks) and an opioid taper (4-weeks) with 8 weeks of post-medication follow-up (95). Few study participants (7%) stopped using other opioids during the brief stabilization and taper; with the extended stabilization and taper, 49% ceased the use of other opioids at the completion of the taper (95). By the end of the 8-week follow-up period, most had returned to opioid use (8% remained opioid free). Long-term outcomes, however, were more favorable. Telephone interviews conducted at 42 months after entering the study found 32% of participants opioid free and not on opioid agonist therapy, 29% were stabilized on opioid agonist therapy and did not meet criteria for an opioid use disorder, 8% were on opioid agonist therapy while continuing to use other opioids, and 31% continued to use opioids and were not on opioid agonist therapy (96). In brief, most individuals with an opioid use disorder have difficulty completing an opioid taper, and a return to opioid use is common.

## TREATMENT RECOMMENDATIONS AND MODELS OF CARE

Persons with an opioid use disorder are at elevated risk of returning to use following opioid cessation, and, because after a period of nonuse their tolerance for opioids subsides, they are at elevated risk for an opioid overdose and death. The evidence-based treatment recommendation for opioid use disorder is either stabilization on opioid agonist therapy or opioid antagonist therapy as a relapse prevention strategy. The American Society of Addiction Medicine’s (ASAM) *National Practice Guideline for the Use of Medications in the Treatment of Addiction Involving Opioid Use* recommends basing the treatment choice, as with medical decision making for other health conditions, on a shared decision-making process between the patient and clinician that includes the consideration of patient preference, treatment history, and treatment setting, as well as an assessment of the patient’s environment, co-occurring disorders, and the risk of buprenorphine diversion (47). Opioid agonist therapy using methadone is appropriate for patients who benefit from the structure of daily dosing in an opioid treatment program or those who have not responded well in outpatient care with buprenorphine. Office-based treatment using buprenorphine as the agonist therapy is appropriate for patients with more structure in their lives but is not appropriate for patients with a co-occurring “alcohol use disorder or sedative, hypnotic or anxiolytic use disorders” (47, p. 361). The ASAM recommendations also note that poor medication adherence is often a problem when using oral naltrexone and that extended-release naltrexone improves medication adherence for patients seeking opioid antagonist therapy (47). Internationally, the World Health Organization (WHO) and the United Nations Office on Drugs and Crime (UNODC) (89) recommend and encourage long-term opioid agonist therapy. The 2017 British Columbia provincial *Guideline for the Clinical Management of Opioid Use Disorder* discourages withdrawing patients from opioids without continued care because of the increased risk of a return to use and potential for a fatal overdose; their first-line recommendation is to use buprenorphine over methadone because of the enhanced safety profile as compared with methadone (80).

A review of opioid use disorder treatment in the primary care setting identified models of care that varied primarily on the basis of how they were staffed, networked, and supported (56). Many DATA-waivered physicians provide office-based opioid treatment in primary care settings prescribing buprenorphine, initiating the medication induction phase for patients and monitoring response with periodic visits, urine drug screening, and prescription refills with referrals for counseling and other support services (28, 29, 30, 31). Most models of care are a variation on this general strategy. The Buprenorphine HIV Evaluation and Support (BHIVES) model focused on providing office-based opioid treatment within the context of HIV primary care (32). The Vermont hub-and-spoke model (11) and Maryland’s collaborative opioid prescribing model (86) are more elaborate variations that link opioid treatment and primary care; patients are stabilized on buprenorphine within an opioid treatment program and, when stable, are referred to physicians and clinics in their communities, where they continue to receive ongoing monitoring and prescription renewals. More complex patients may remain connected or return to the opioid treatment program for intensive addiction treatment services and monitoring as needed. The Massachusetts nurse care manager model adds a nurse care manager to facilitate patient management and to reduce burdens on prescribers (6, 59). Project Extension for Community Healthcare Outcomes (Project ECHO) provides ongoing support for office-based practices through telemedicine (55). The Oregon Pain Guidance Group emerged organically and supports medical practices to address pain and opioid use disorders in an integrated approach to care; resources include (*a*) a website with tools for patients and practitioners, (*b*) an annual conference, and (*c*) opioid-prescribing guidelines (https://professional.oregonpainguidance.org/). Only a minority of the care models have been tested and validated in clinical trials, and no studies compare the relative effectiveness of the different models (56). The current models focus on the use of buprenorphine as an opioid agonist therapy but could easily be used for opioid antagonist therapy.

## PREVENTION OF OPIOID USE DISORDER

A sevenfold increase in fatalities attributed to opioid overdose (from ~4,000 deaths per year in 1999 to 28,647 in 2014 and 33,091 in 2015) (83, 94), the introduction of illicitly manufactured fentanyl and fentanyl analogs (14), and a spike in opioid overdoses related to fentanyl (76, 85) sparked federal and local initiatives to reduce opioid misuse and to prevent overdoses. Project Lazarus, developed in 2007, addressed overdose mortality in Wilkes County, North Carolina, with systematic attention to provider education, emergency department policies, diversion control, pain support, harm reduction, addiction treatment, and community education combined with collection and monitoring of indicator data and coalition action (5, 79). Oregon’s Opioid Taskforce recommended naloxone distribution and overdose education, increased access to opioid agonist therapy, strategies for safe disposal of unused opioids, modification to the prescription drug monitoring program to promote routine use prior to prescribing opioids, and encouraged adherence to opioid prescribing guidelines (64).

The Office of National Drug Control Policy (ONDCP), the Department of Health and Human Services, and the US Surgeon General developed strategies to address the opioid epidemic. The 2011 Prescription Drug Abuse Prevention Plan expanded on the Obama Administration’s *National Drug Control Strategy* and included four action steps to reduce prescription opioid misuse (71). A crucial first step in tackling the problem of opioid abuse is to educate parents, youth, and patients about the dangers of misusing medications, while requiring prescribers to receive education on the appropriate and safe use and proper storage and disposal of opioid medications. The plan also promoted implementation of prescription drug monitoring programs (PDMPs) in every state to reduce “doctor shopping” and diversion and to increase state data sharing and prescriber use. Every state (including Guam and the District of Columbia) except for Missouri has operational PDMPs, and on July 17, 2017, the Governor of Missouri signed an executive order authorizing the Missouri PDMP (70). PDMPs vary in the operational agency (e.g., the pharmacy board, law enforcement), specific rules of use (e.g., who is required to use the PDMP), data-sharing policies (e.g., who is allowed to view the data), and data collection processes (e.g., how often) (21, http://www.pdmpassist.org/). The increased availability of unused pills from opioid prescriptions contributes to nonmedical use of opioids, in part because of DEA restrictions on medication disposal or return; DEA regulations were amended in 2014 to permit convenient and environmentally responsible prescription drug disposal programs to help decrease the supply of unused prescription drugs in the home. The ONDCP also sought to provide law enforcement with the tools necessary to eliminate improper prescribing practices.

Within the Department of Health and Human Services, the Office of the Assistant Secretary for Planning and Evaluation supported the ONDCP Prescription Drug Strategy and targeted three priority areas: prescribing practices, naloxone distribution, and access to opioid agonist and antagonist therapy (69a). In 2016, the Centers for Disease Control and Prevention (CDC) published the *CDC Guideline for Prescribing Opioids for Chronic Pain* (25, 26), which prioritized the use of nonopioid analgesics for chronic pain treatment, encouraged the use of the lowest effective dose of opioids when necessary, suggested caution when prescribing 50 or more morphine milligram equivalents (MME) per day, and discouraged prescriptions of 90 or more MMEs per day. The US Surgeon General sent a letter to 2.3 million opioid prescribers to urge them to adopt and adhere to the *CDC Guideline* (67) and released the first Surgeon General’s Report on alcohol, drugs, and health (73). All the federal initiatives sought to reduce the availability of opioid medications and to reduce the potential for lethal overdoses. The rates of opioid overdoses, however, have not yet begun to reverse.

To advance the federal role in the prevention and treatment of opioid use disorders, President Donald Trump issued an Executive Order establishing the President’s Commission on Combating Drug Addiction and the Opioid Crisis (97). New Jersey Governor Chris Christie chairs the Commission, and membership includes the US Attorney General, the Secretary of Health and Human Services, the Secretary of Veterans Affairs, the Secretary of Defense, and five additional members named from state government, law enforcement, and other stakeholders. The Commission will assess the availability of drug treatment services, identify best prevention practices, assess the effectiveness of provider education and addiction prevention, evaluate existing federal programs, and recommend program improvements (98).

## OVERDOSE EDUCATION AND NALOXONE DISTRIBUTION

The FDA approved the opioid antagonist naloxone (Narcan®) to counteract the respiratory depression associated with opioid overdose in 1971. Use was initially restricted to emergency responders and emergency departments and administered as an intravenous or intramuscular injection. As access to prescription opioids increased in the 1990s and the early decades of the twenty-first century, the need for naloxone rescue increased and community activists began overdose education and naloxone distribution programs. Overdose education teaches participants (e.g., patients, family, friends, community members) to recognize opioid overdose symptoms and to administer naloxone (48). States are permitting the distribution of naloxone through pharmacies and allowing physicians to prescribe for concerned others and family members. An interrupted time series analysis of opioid overdose fatality rates in 19 Massachusetts communities documented a decline in the risk of opioid overdose fatalities following the introduction of overdose education and naloxone distribution; the decline was greater in communities where more people were trained (93). To facilitate access to naloxone, the FDA approved autoinjector (in 2014) and nasal spray formulations (in 2015), and the number of naloxone prescriptions increased slightly from 2.8 million (in 2009) to 3.2 million (in 2015) (36). Prices have increased distressingly; the 2016 price of the autoinjection formulation was $4,500 versus the 2014 price of $690 (36).

## INTERNATIONAL TREATMENT OPTIONS

Despite having access to evidence-based treatment, persons with opioid use disorder often continue to use opioids. Some European countries and Canadian provinces offer a greater array of treatment and harm-reduction interventions. For patients who are refractory to buprenorphine or methadone treatment and continue to use non-treatment-based opioids, the British Columbia provincial guidelines recommend that patients and physicians consider slow-release oral morphine if prescribed as a once daily witnessed dose (80). Vancouver, British Columbia, hosts the only medically supervised injection clinic in North America. The Crosstown Clinic provides medical-grade heroin (diacetylmorphine) or hydromorphone for injection three times daily for patients with severe treatment-refractory opioid use disorder. European and Canadian studies document the value of opioid agonist therapy using diacetylmorphine (9, 49, 69, 75). Vancouver also hosts North America’s first supervised injection facility (called Insite), which provides clean equipment for injecting, a safe and clean facility, and a medically supervised environment to promote safer injection of opioids and other drugs (100, 101). Evaluations suggest that Insite contributed to reductions in needle sharing, overdoses, and fatal overdoses and increased treatment referrals (19, 50, 63).

## DISCUSSION

The Lexington and Fort Worth Hospitals provided the initial US opioid and drug use disorder treatment framework. A 1990 review of drug treatment noted that the Public Health Hospitals were significant because they (*a*) served both inmates and volunteers, (*b*) were the first allocation of federal funds for specialized drug treatment services, and (*c*) trained clinicians and investigators to address opioid and other drug use disorders (10, 39). NARA extended access to care through the authorization of and funding for community-based outpatient care. Methadone services research documented the efficacy of agonist therapy, and the development of buprenorphine and extended-release naltrexone permitted office-based treatment for opioid use disorder.

Federal policy has shaped how opioid use disorder is treated and accessed. In the past decade, four sets of federal legislation and Medicaid regulations continued to shape opioid use disorder treatment. The 2008 Mental Health Parity and Addiction Equity Act (MHPAEA) required group and individual health plans to administer equitable benefits for mental health and substance use disorders as compared with medical/surgical benefits; more restrictive quantitative (e.g., amounts covered) and qualitative (e.g., preauthorization and utilization management) limits are prohibited (89a). In theory, there should be fewer barriers to accessing care. The regulations implementing MHPAEA were effective for most plans in July 2014, and it is unclear if access to treatment for mental health and substance use disorders has improved.

The 2010 Patient Protection and Affordable Care Act (ACA) was designed to reduce the number of uninsured persons and create state and federal insurance exchanges through which uninsured individuals could purchase insurance at discounted rates based on income. Tax incentives to the health insurance providers offset the reduced cost of the insurance. The ACA allowed states to expand eligibility for state Medicaid plans and identified benefits for mental health and substance use disorders as essential benefits that must be included in plans offered under the ACA. The ACA promoted integration of care for alcohol and drug use disorders. Observers anticipated that expanded access to health care and health insurance would facilitate entry into treatment for opioid use disorder (3, 8, 12). Congress, however, continues to seek the repeal and replacement of the ACA. The future is unclear, and some experts in addiction treatment and policy assert that weakening or repealing the ACA could worsen the opioid epidemic because the ACA expanded Medicaid eligibility, enhanced access to lower-cost health insurance, and required health plans to include treatment for mental health and substance use disorders as essential benefits (33).

In 2015 and 2016, the Centers for Medicare and Medicaid Services (CMS) reduced restrictions on the use of Medicaid to purchase residential services for treatment of opioid use disorder and other substance use disorders. Medicaid has not historically covered residential services in inpatient programs with more than 16 beds for mental health and substance use disorders because the programs were classified as institutions for mental disease (known as the IMD exclusion). A July 2015 letter from the CMS Director to State Medicaid Directors announced an opportunity to apply for a Section 1115 demonstration waiver to redesign state substance use treatment systems (92). States receiving waiver approval are exempt from the IMD exclusion and are permitted to use Medicaid funds to purchase limited inpatient and residential services for treating substance use disorders, including opioid use disorder. Waiver approval requires development of a comprehensive continuum of care, including evidence-based services for screening, brief intervention, referral to care, pharmacotherapy for alcohol and opioid use disorders, short-term institutional services (i.e., residential care), and ongoing recovery services. To control the use of institutional care, states are required to use the ASAM patient placement (7) and to have an independent third party conduct the assessment. As of October 13, 2017, 5 states had approved 1115 waivers from the IMD exclusion: California, Maryland, Massachusetts, New York (90) and West Virginia (90a). Seven states have waiver applications pending: Arizona, Illinois, Indiana, Kentucky, Michigan, Utah, and Virginia (90). The IMD exclusion was also relaxed in CMS’s Medicaid and CHIP Managed Care Final Rule (15). The regulations permitted plans to continue to receive monthly capitation payments for patients receiving care in an IMD if the services were an alternative to more expensive care and were specifically treating psychiatric or substance use disorders (15).

Two more pieces of federal legislation addressed the opioid epidemic in 2016: the Comprehensive Addiction Recovery Act (CARA; May 2016) (P.L. 114–198) and the 21st Century Cures Act (December 2016) (P.L. 114–255). CARA expanded prevention and public education on opioids and other substance use disorders, enhanced availability of naloxone for law enforcement and other first responders to reverse opioid overdoses, promoted access to opioid agonist and antagonist therapy for incarcerated individuals, authorized physician assistants and nurse practitioners to prescribe buprenorphine, expanded disposal options for unused medications, authorized grants to states for evidence-based opioid treatment and intervention demonstrations, and strengthened state PDMPs (15a). The 21st Century Cures Act allocated $1 billion to support the state demonstrations authorized in CARA

Opioid prevention and treatment efforts in the United States remain constrained and consequently stigmatized by a legacy of federal restrictions, an unwillingness to acknowledge idiopathic addiction, and a lack of science-based interventions for chronic pain. In July 2017, the Director of the National Institutes of Health and the Director of the National Institute on Drug Abuse announced a public/private research partnership to develop medications and technologies in three areas to prevent opioid-related mortality and promote recovery: (*a*) reduction in overdose fatalities, (*b*) enhancement of treatment for opioid use disorders, and (*c*) development of nonaddictive medications and interventions for chronic pain (91). Over the next decade, we expect to see enhanced access to opioid agonist and antagonist therapy, improved integration with primary care, and a reduction in access to and the use of opioids for the treatment of chronic non-cancer pain. The lessons learned in the first two decades of the new century have been hard learned and painful. Systems of prevention and care need to embrace evidence-based therapies and require integrated primary and specialty care to better serve patients struggling with opioid use disorder.

## References

[R1] 121st Century Cures Act, Pub. L. No. 114–255, 130 Stat. 1033 (2016). https://www.congress.gov/bill/114th-congress/house-bill/6/

[R2] 2Deleted in proof

[3] Abraham AJ, Andrews CM, Grogan CM, D’Aunno T, Humphreys KN (2017). The Affordable Care Act transformation of substance use disorder treatment. Am J Public Health.

[4] Alanis-Hirsch K, Croff R, Ford JH, Johnson K, Chalk M (2016). Extended-release naltrexone: a qualitative analysis of barriers to routine use. J Subst Abuse Treat.

[5] Albert S, Brawon FW, Sanford CK, Dasgupta N, Graham J, Lovette B (2011). Project Lazarus: community-based overdose prevention in rural North Carolina. Pain Med.

[6] Alford DP, LaBelle CT, Kretsch N, Bergeron A, Winter M (2011). Collaborative care of opioid-addicted patients in primary care using buprenorphine: five-year experience. Arch Intern Med.

[7] Mee-Lee D, ASAM (Am. Soc. Addict. Med.) (2013). The ASAM Criteria: Treatment Criteria for Addictive, Substance-Related, and Co-Occurring Conditions.

[8] Barry CL, Huskamp HA (2011). Moving beyond parity—mental health and addiction care under the ACA. N Engl J Med.

[9] Berridge V (2009). Heroin prescription and history. N Engl J Med.

[10] Besteman KJ, Gerstein DR, Harwood HJ (1992). Federal leadership in building the national drug treatment system. Treating Drug Problems.

[11] Brooklyn JR, Sigmon SC (2017). Vermont hub-and-spoke model of care for opioid use disorder: development, implementation, and impact. J Addict Med.

[12] Buck JA (2011). The looming expansion and transformation of public substance abuse treatment under the Affordable Care Act. Health Aff.

[13] Campbell ND, Olsen JP, Walden L (2008). The Narcotic Farm: The Rise and Fall of America’s First Prison for Drug Addicts.

[14] CDC (Cent. Dis. Control Prev.) Health Advis (2015). Increases in fentanyl drug confiscations and fentanyl-related overdose fatalities CDCHAN-00384.

[15] CMS (Cent. Medicare Medicaid Serv.) (2016). Medicaid and CHIP managed care final rule. Fed Regist.

[R16] 15aComprehensive Addiction and Recovery Act of 2016, Pub. L. 114–198, 130 Stat. 695 (2016). https://www.congress.gov/bill/114th-congress/senate-bill/524/text

[16] Courtwright D, Joseph H, Des Jarlais D (1989). Addicts Who Survived: An Oral History of Narcotic Use in America, 1923–1965.

[17] Courtwright DT (1982). Dark Paradise: Opiate Addiction in America Before 1940.

[18] De Leon G, Rosenthal MS (1979). Therapeutic communities.

[19] DeBeck K, Kerr T, Bird L, Zhang R, Marsh DC (2011). Injection drug use cessation and use of North America’s first medically supervised safer injecting facility. Drug Alcohol Depend.

[R21] 20Deleted in proof

[21] Deyo RA, Irvine JM, Millet LM, Beran T, O’Kane N (2013). Measures such as interstate cooperation would improve the efficacy of programs to track controlled drug prescriptions. Health Aff.

[22] Divers. Control Div (2017). Controlled substance schedules.

[23] Dole VP, Nyswander M (1965). A medical treatment of diacetylmorphine (heroin) addiction: a clinic trial with methadone hydrochloride. JAMA.

[24] Dole VP, Nyswander ME, Kreek MJ (1966). Narcotic blockade. Arch Intern Med.

[25] Dowell D, Haegerich TM, Chou R (2016). CDC guideline for prescribing opioids for chronic pain—United States, 2016. JAMA.

[26] Dowell D, Haegerich TM, Chou R (2016). CDC guideline for prescribing opioids for chronic pain—United States, 2016. MMWR.

[27] Drug Enforc. Adm. Off. Divers. Control (2006). Practitioner’s Manual: An Informational Outline of the Controlled Substances Act.

[27a] Dupont RI, Goldstein A, O’Donnell J (1979). Handbook on Drug Abuse.

[28] Fiellin DA, Moore BA, Sullivan LE, Becker WC, Pantalon MV (2008). Long-term treatment with buprenorphine/naloxone in primary care: results at 2–5 years. Am J Addict.

[29] Fiellin DA, O’Conner PG (2002). Office-based treatment of opioid-dependent patients. N Engl J Med.

[30] Fiellin DA, Pantalon MV, Chawarski MC, Moore BA, Sullivan LE (2006). Counseling plus buprenorphine-naloxone maintenance therapy for opioid dependence. N Engl J Med.

[31] Fiellin DA, Pantalon MV, Pakes JP, O’Conner PG, Chawarski MC, Schottenfeld RS (2002). Treatment of heroin dependence with buprenorphine in primary care. Am J Drug Alcohol Abuse.

[32] Fiellin DA, Weiss L, Botsko M, Egan JE, Altice FL (2011). Drug treatment outcomes among HIV-infected opioid-dependent patients receiving buprenorphine/naloxone. J Acquir Immune Defic Syndr.

[33] Friedmann PD, Andrews CM, Humphreys K (2017). How ACA repeal would worsen the opioid epidemic. N Engl J Med.

[34] Gastfriend DR (2011). Intramuscular extended-release naltrexone: current evidence. Ann N Y Acad Sci.

[35] Gourevitch MN, Arnsten J, Lowinson JH, Ruiz P, Millman RB, Langrod JG (2005). Medical complications of drug use. Substance Abuse: A Comprehensive Textbook.

[36] Gupta R, Shah ND, Ross JS (2016). The rising price of naloxone—risks to efforts to stem overdose deaths. N Engl J Med.

[37] Hser YI, Hoffman V, Grella CE, Anglin MD (2001). A 33-year follow-up of narcotic addicts. Arch Gen Psychiatry.

[38] Hughes A, Williams MR, Lipari RN, Bose J, Copello EAP, Kroutil LA (2016). Prescription drug use and misuse in the United States: results from the 2015 National Survey on Drug Use and Health. NSDUH Data Rev.

[39] Gerstein DR, Harwood HJ, Inst. Med (1990). Treating Drug Problems. A Study of the Evolution, Effectiveness, and Financing of Public and Private Drug Treatment Systems.

[40] Retting RA, Yarmolinsky A, Inst. Med (1995). Federal Regulation of Methadone Treatment.

[41] Edmunds M, Frank R, Hogan M, McCarty D, Robinson-Beale R, Weisner C, Inst. Med (1997). Managing Managed Care: Quality Improvement in Behavioral Health.

[42] Jaffe JH (1997). The history and current status of opiate agonist treatment.

[43] Jaffe JH, O’Keeffe C (2003). From morphine clinics to buprenorphine: regulating opioid agonist treatment of addiction in the United States. Drug Alcohol Depend.

[44] Jasinski DR, Pevnick JS, Griffith JD (1978). Human pharmacology and abuse potential of the analgesic buprenorphine: a potential agent for treating narcotic addiction. Arch Gen Psychiatry.

[45] Joseph H, Stancliff S, Langrod J (2000). Methadone maintenance treatment (MMT): a review of historical and clinical issues. Mt Sinai J Med.

[46] Julius D, Julius D, Renault P (1976). NIDA’s naltrexone research program. Narcotic Antagonists: Naltrexone. Progress Report. NIDA Res Monogr 9.

[47] Kampman K, Jarvis M (2015). American Society of Addiction Medicine (ASAM) National Practice Guideline for the use of medications in the treatment of addiction involving opioid use. J Addict Med.

[48] Kerensky T, Walley AY (2017). Opioid overdose prevention and naloxone rescue kits: what we know and what we don’t know. Addict Sci Clin Pract.

[49] Kerr T, Montaner JSG, Wood E (2010). Science and politics of heroin prescription. Lancet.

[50] Kerr T, Tyndall MW, Li K, Montaner J, Wood E (2005). Safer injection facility use and syringe sharing in injection drug users. Lancet.

[51] Knudsen HK, Abraham AJ, Oser CB (2011). Barriers to the implementation of medication-assisted treatment for substance use disorders: the importance of funding policies and medical infrastructure. Eval Prog Plan.

[52] Knudsen HK, Abraham AJ, Roman PM (2011). Adoption and implementation of medications in addiction treatment programs. J Addict Med.

[53] Knudsen HK, Roman PM (2012). Financial factors and the implementation of medications for treating opioid use disorders. J Addict Med.

[54] Kolodny A, Courtwright DT, Hwang CS, Kreiner P, Eadie JL (2015). The prescription opioid and heroin crisis: a public health approach to an epidemic of addiction. Annu Rev Public Health.

[55] Komaromy M, Duhigg D, Metcalf A, Carlson C, Kalishman S (2016). Project ECHO (Extension for Community Healthcare Outcomes): a new model for educating primary care providers about treatment of substance use disorders. Subst Abuse.

[56] Korthuis PT, McCarty D, Weimer M, Bougatsos C, Blazina I (2017). Primary care-based models for the treatment of opioid use disorder: a scoping review. Ann Intern Med.

[57] Kreek MJ, Vocci FJ (2002). History and current status of opioid maintenance treatments. J Subst Abuse Treat.

[58] Krupitskii E, Nunes EV, Ling W, Illeperuma A, Gastfriend DR, Silverman BL (2011). Injectable extended-release naltrexone for opioid dependence: a double-blind, placebo-controlled, multicenter randomised trial. Lancet.

[59] LaBelle CT, Han SC, Bergeron A, Samet JH (2016). Office-based opioid treatment with buprenorphine (OBOT-B): statewide implementation of the Massachusetts collaborative care model in community health centers. J Subst Abuse Treat.

[60] Legisetty P (2017). The fine line between doctoring and dealing. Health Aff.

[61] Leukefeld CG, Pickens RW, Leukefeld CG, Schuster CR (1991). Opportunities for enhancing drug abuse treatment with criminal justice authority. Improving Drug Abuse Treatment.

[62] Maddux JF, Leukefeld CG, Tims FM (1988). Clinical experience with civil commitment. Compulsory Treatment of Drug Abuse: Research and Clinical Practice.

[63] Marshall BDL, Milloy M-J, Wood E, Montaner JSG, Kerr T (2011). Reduction in overdose mortality after the opening of North America’s first medically supervised safer injecting facility: a retrospective population-based study. Lancet.

[64] McCarty D, Bovett R, Burns T, Cushing J, Glynn ME (2015). Oregon’s strategy to confront prescription opioid misuse: a case study. J Subst Abuse Treat.

[65] Minist. Health (1926). Rolleston Report: Departmental Committee on Morphine and Heroin Addiction.

[66] Minozzi S, Amato L, Vecchi S, Davoli M, Kirchmayer U, Verster A (2011). Oral naltrexone maintenance treatment for opioid dependence. Cochrane Database Syst Rev.

[67] Murthy VH (2016). Ending the opioid epidemic—a call to action. N Engl J Med.

[68] Musto DF (1973). The American Disease: Origins of Narcotic Control.

[69] Nosyk B, Guh DP, Bansback NJ, Oviedo-Joekes E, Brissette S (2012). Cost-effectiveness of diacetylmorphine versus methadone for chronic opioid dependence refractory to treatment. Can Med Assoc J.

[69a] Off. Assist. Secr. Plan. Eval (2015). Opioid abuse in the U.S. and HHS actions to address opioid-drug related overdoses and deaths. ASPE Issue Brief.

[70] Off. Mo. Gov. Eric Greitens (2017). Governor Eric Greitens announces statewide prescription drug monitoring program. News Release.

[71] Off. Natl. Drug Control Policy (2011). Epidemic: Responding to America’s Prescription Drug Abuse Crisis.

[R75] 72Deleted in proof

[73] Off. Surg. Gen (2016). Facing Addiction in America: The Surgeon General’s Report on Alcohol Drugs and Health.

[R77] 74Deleted in proof

[75] Oviedo-Joekes E, Brissette S, Marsh DC, Lauzon P, Guh D (2009). Diacetylmorphine versus methadone for the treatment of opioid addiction. N Engl J Med.

[76] Peterson AB, Gladden RM, Delcher C, Spies E, Garcia-Williams A (2016). Increases in fentanyl-related overdose deaths—Florida and Ohio 2013–2015. MMWR.

[77] Phillips KA, Friedmann PD, Saitz R, Samet JH (2014). Linking addiction treatment with other medical and psychiatric treatment systems.

[R81] 78Deleted in proof

[79] Proj. Lazarus (2017). The Project Lazarus model.

[80] Prov. Opioid Use Disord. Treat. Guidel. Comm (2017). A Guideline for the Clinical Management of Opioid Use Disorder.

[81] Resnick RB, Schuyten-Resnick E, Washton AM (1979). Treatment of opioid dependence with narcotic antagonists.

[82] Rieder TN (2017). In opioid withdrawal, with no help in sight. Health Aff.

[82a] Ries RK, Fiellin DA, Miller SC, Saitz R (2014). The ASAM Principles of Addiction Medicine.

[83] Rudd RA, Seth P, David F, Scholl L (2016). Increases in drug and opioid-involved overdose deaths—United States, 2010–2015. MMWR.

[84] Saitz R (2014). Medical and surgical complications of addiction.

[84a] SAMHSA (Subst. Abuse Mental Health Serv. Adm.) (2017). National Survey of Substance Abuse Treatment Services (N-SSATS): 2016. Data on Substance Abuse Treatment Facilities.

[84b] SAMHSA (Subst. Abuse Mental Health Serv. Adm.) (2017). Treatment Episode Data Set (TEDS): 2005–2015. National Admissions to Substance Abuse Treatment Services.

[85] Somerville NJ, O’Donnell J, Gladden RM, Zibbell JE, Green TC (2017). Characteristics of fentanyl overdose—Massachusetts, 2014–2016. MMWR.

[86] Stoller KB (2015). A collaborative opioid prescribing (CoOP) model linking opioid treatment programs with office-based buprenorphine providers. Addict Sci Clin Pract.

[R93] 87Deleted in proof

[R94] 88Deleted in proof

[89] UNODC (UN Off. Drugs Crime), WHO (World Health Organ.) (2017). International Standards for the Treatment of Drug Use Disorders.

[89a] US Dep. HHS (Health Hum. Serv.) (2016). The Mental Health and Substance Use Disorder Parity Task Force: Final Report.

[90] Vestal C (2017). States seek Medicaid dollars for addiction treatment beds. Stateline.

[90a] Centers for Medicare & Medicaid Services (2017). Dear Ms Beane.

[91] Volkow ND, Collins FS (2017). The role of science in addressing the opioid crisis. N Engl J Med.

[92] Wachino V (2015). Re: New service delivery opportunities for individuals with a substance use disorder.

[93] Walley AY, Xuan Z, Hackman HH, Quinn E, Doe-Simkins M (2013). Opioid overdose rates and implementation of overdose education and nasal naloxone distribution in Massachusetts: interrupted time series analysis. BMJ.

[94] Warner M, Chen LH, Makuc DM, Anderson RN, Miniño AM (2011). Drug poisoning deaths in the United States, 1980–2008. NCHS Data Brief.

[95] Weiss RD, Potter JS, Fiellin DA, Byrne M, Connery HS (2011). Adjunctive counseling during brief and extended buprenorphine-naloxone treatment for prescription opioid dependence: a 2-phase randomized controlled trial. Arch Gen Psychiatry.

[96] Weiss RD, Potter JS, Griffin ML, Provost SE, Fitzmaurice GM (2015). Long-term outcomes from the National Drug Abuse Treatment Clinical Trials Network Prescription Opioid Addiction Treatment Study. Drug Alcohol Depend.

[96a] White W, Callahan JF (2014). Addiction medicine in America: its birth and early history (1750–1935) with a modern postscript.

[97] White House Off. Press Secr (2017). President Donald J. Trump signs an Executive Order establishing the President’s Commission on Combating Drug Addiction and the Opioid Crisis. Press Release.

[98] White House Off. Press Secr (2017). Presidential Executive Order establishing the President’s Commission on Combating Drug Addiction and the Opioid Crisis. News Release.

[R108] 99Deleted in proof

[100] Wood E, Kerr T, Lloyd-Smith E, Buchner C, Marsh DC (2004). Methodology for evaluating Insite: Canada’s first medically supervised safer injection facility for injection drug users. Harm Reduct J.

[101] Wood E, Kerr T, Small W, Li K, Marsh DC (2004). Changes in public order after the opening of a medically supervised safer injecting facility for illicit injection drug users. Can Med Assoc J.

